# Oral health related quality of life among HIV positive patients attending two HIV outpatient clinics in Nigeria - a cross sectional study

**DOI:** 10.4314/ahs.v21i2.11

**Published:** 2021-06

**Authors:** Kehinde Adesola Umeizudike, Babatope Bamidele Osagbemiro, Opeyemi Oluwayemisi Daramola, Titilope Adenike Adeyemo

**Affiliations:** 1 Department of Preventive Dentistry, Faculty of Dental Sciences, College of Medicine, University of Lagos, Idi-Araba, Lagos State. kumeiz09@gmail.com; 2 Department of Preventive Dentistry, University of Port-Harcourt Teaching Hospital, Rivers State. topegraphics03@yahoo.co.uk; 3 Department of Preventive Dentistry, Lagos University Teaching Hospital, Idi Araba, Lagos State. opeyemidaramola1985@gmail.com; 4 Department of Haematology & Blood transfusion, College of Medicine, University of Lagos, Idi-Araba, Lagos State. taadeyemo@cmul.edu.ng

**Keywords:** HIV infection, Oral health, OHRQoL

## Abstract

**Background:**

The human immunodeficiency virus infection remains a devastating disease of public health importance.

**Objectives:**

To assess the association between oral health and quality of life and the factors affecting the oral health related quality of life among HIV positive patients in Nigeria.

**Methods:**

This was a cross sectional study of HIV positive patients attending two HIV outpatient clinics in Nigeria. Impact of oral health on quality of life was assessed using the OHIP-14. Oral health status was assessed by the DMFT and Simplified OHI indices. Level of significance was set at p< 0.05.

**Results:**

Three hundred and fifty-two patients were seen, 64.2% being females. Prevalence of impact was 8.5%; and the mean OHIP scores was 8.05±9.54. Highest impact was “painful aching” 67(19.1%) with the domain of physical pain scoring the highest mean impact of 2.32. Most patients (88.6%) were on HAART. Following logistic regression, after controlling for potential confounders, independent factors associated with poor OHRQoL were perceived need for dental treatment, HAART use, and higher DMFT (p<0.05).

**Conclusion:**

The domain of physical pain had the highest impact, while perceived need for dental treatment, HAART use and higher caries index were contributory to poor OHRQoL.

## Introduction

The human immunodeficiency virus (HIV) infection has been one of the most devastating diseases of public health importance affecting mankind in the past few decades. Not only has the virus infected a huge population of people (estimated to be 75 million) globally since the beginning of the pandemic, it has also resulted in the death of at least 32 million people from Acquired Immunodeficiency syndrome (AIDS)-related illnesses^1^. Currently, the number of HIV infections stands at about 37.9 million people^1^ with a global prevalence of 0.8% among adults^2^. The vast majority of people living with HIV/AIDS (PLWHA) reside in low- and middleincome countries^3^. The epicentre of the HIV infection is sub-Saharan Africa, which bears most of the burden, affecting approximately 68%, which is an estimated 25.7 million people^4^. This is alarming because of the restricted resources in Africa where the HIV infection is one of the leading causes of mortality.

The current prevalence rate of HIV in Nigeria is 1.4%, which is lower than the 3.9% previously reported in 2008^5^. This has been attributed to better surveillance and access to antiretroviral treatment^5^. Despite the lower rate, Nigeria still has the largest number of PL-WHA in Africa and has the second largest HIV epidemic globally due to huge number of people infected, an estimated 1.9 million (adults aged 15–49 years)^5^. HIV infection thus contributes to the burden of disease and remains a significant public health threat for Nigerians^5^. The quality of life of PLWHA may be impacted by factors such as poor oral health, which has been known to be a contributing factor to the development of opportunistic infections in persons living with HIV/AIDS^6^. Oral manifestations of HIV infection are well established in the literature and may occur in over 50% of HIV/AIDS patients^7^. These oral lesions often present as early signs and symptoms of the disease and most commonly include HIV gingivitis and periodontitis, fungal and other opportunistic infections as well as Kaposi's sarcoma^7^,^8^,^9^. Whilst, lesions such as candidiasis and hairy leukoplakia are considered as prognostic factors of HIV infection, reports indicate that concurrent existence of multiple and variable oral lesions is accompanied by poor prognosis of HIV infection^10^.

The advent of antiretroviral therapy (ART) particularly the highly active antiretroviral therapy (HAART) has improved the quality of life of HIV positive patients and has reduced to a very large extent the prevalence of oral lesions in HIV infected patients^11^,^12^. A lower prevalence of some oral lesions have also been reported in Nigeria^13^. A reduced rate of salivary flow has been reported on PLWHA on HAART which may be related to the type of ART^14^. Improved access to ART has significantly increased life expectancy for HIV positive persons^15^. The scale-up HIV response by the Nigerian government to include about 1 million patients on HAART across 1500 treatment sites is encouraging^5^, and would no doubt prolong the life expectancy of PLWH in Nigeria. However, some oral lesions in HIV infection appear to predict disease progression and may signify the failure of HAART and can substantially contribute to deterioration in the quality of life of HIV infected patients^16^.

The HIV-1 infection has an independent and negative impact on the oral HRQoL^17^. The impact of HIV/AIDS and oral disease on the Quality of Life affects physical and emotional well-being, social support systems, and life roles^7^,^18^. A variety of instruments have been developed to measure the oral health quality of life^19^. The Oral Heath Impact Profile (OHIP), in both the long (OHIP-49) and short forms (OHIP-14), is one of the most commonly used instruments to evaluate the impact of different oral disorders on well-being. It was developed by Slade and Spencer in 1994 and contains seven dimensions that are based on Locker's theoretical model of oral health^20^. The dimensions are: functional limitation, physical pain, psychological discomfort, physical disability, psychological disability, social disability and handicap^21^. Patients with more severe AIDS manifestations had more oral symptoms, functional limitations, emotional and social well being related to their oral health^18^. HIV-positive patients with some oral lesions experienced greater social impacts which were associated with poorer oral health related quality of life^22^. In addition to the influence of social class, the perception of the influence of oral health on the quality of life was also shown to be influenced by age and sex in the HIV positive patients^7^. A poorly functioning dentition can complicate the management of medical conditions and create or exacerbate nutritional and psychosocial problems. Oral health care may improve the health related quality of life in HIV/AIDS patients^23^.

As people are living longer with HIV, the study on their quality of life has emerged as an important factor in their overall management^24^. This was also highlighted by the Centers for Disease Control and Prevention (CDC) whose goal is to improve health outcomes of PLWHA by extending life and its quality^25^. Although, several studies have been carried out on oral lesions among HIV positive patients with and without HAART therapy in Nigeria^8^,^9^,^16^, to the best of our knowledge, there is no published study on the oral health related quality of life (OHRQoL) among PLWHA in Nigeria. The aim of this study was therefore to determine the association between oral health and the quality of life and the factors affecting OHRQoL among HIV positive patients in Nigeria.

## Methodology

This cross-sectional, descriptive study was part of a larger study among HIV positive patients attending two HIV dedicated out-patient clinics. The HIV status of the patients was assessed and confirmed using the WHO recommended HIV testing algorithm. The HIV clinics selected for the study were located at the Lagos University Teaching Hospital (LUTH) [Lagos state, South-west Nigeria] and the University of Port-Harcourt Teaching Hospital (UPTH) (Rivers state, Southsouth Nigeria].

The HIV clinic in LUTH was selected being one of the foremost HIV Treatment centres in Nigeria, coupled with the fact that Lagos is a cosmopolitan state with diverse ethnic representation. The HIV clinic in Rivers state was selected because the state had the highest HIV prevalence rate in Nigeria during the study period.

The participants were recruited into the study between June and October, 2016 to assess the factors associated with their oral health related quality of life. The inclusion criteria were subjects ≥ 17 years, and diagnosis of HIV infection of at least one-year's duration. Patients who were psychologically compromised, had neurological deficit or who refused to give their informed consent were excluded from the study. The questionnaires were kept anonymous and all information collected during the study were treated as confidential. The sample size was calculated using the Fisher's formula for both study centres. A convenience sampling method was used to recruit consecutive subjects who presented for their scheduled outpatient appointment at the HIV clinic into the study until the desired sample size was achieved.

Ethical approval was sought and obtained from the Institutional Review Committees of both institutions before commencing the study. Data was collected with the aid of structured, self-administered questionnaires. Information was obtained on respondents' socio-demographic details, past dental visits, self-rated oral health and self-assessed perceived treatment needs. A detailed medical history was also obtained including their CD4 + T lymphocyte counts, and the length of time since their initial HIV/AIDS diagnosis. The subjects were categorized into different socio-economic status (SES) by combining education and occupation according to Oyedeji^26^. The scores from both categories were added and an average derived by dividing by 2. The subjects were then categorized into upper SES (1), middle SES (2 and 3), and lower SES (4 and 5).

Impact of oral health on quality of life was assessed using questions from the shortened version of the Oral Health Impact Profile (OHIP-14)^20^. The OHIP-14 questionnaire consists of 14 statements that have been rephrased as questions under 7 broad dimensions or domains as follows: functional limitation, physical pain, psychological discomfort, physical disability, psychological disability, social disability and handicap. Subjects were asked to indicate on a five-point Likert scale how frequently they experienced each problem within a reference period (1 year). Response categories for the five-point scale were: “Very often”, “Fairly often”, “Occasionally”, “Hardly ever” and “Never”. Oral examination was carried out under natural lighting by examiners who had been previously calibrated. Oral health status was assessed by the DMFT (Decayed, Missing & Filled Teeth) index and the Simplified Oral Hygiene Index (OHI-S) by Greene & Vermillion^27^. The DMFT index values were graded as Very mild (<5.0), mild (5.0 - 8.9), Moderate (9.0–13.9), severe (>13.9)^28^. Oral lesions associated with HIV/AIDS were documented using the presumptive diagnosis of the EC-Clearinghouse, 1993 guidelines on the diagnostic criteria for classifying oral lesions in HIV^29^.

## Data Analysis

The analysis was done using the SPSS version 20 (IBM SPSS Armonk, New York) software. For data entry, responses on the five-point Likert scale were coded 0 (never), 1(hardly ever), 2(occasionally), 3(fairly often) or 4(very often). The severity of impact in each individual is the sum of all ordinal response codes. The severity may range from 0 to 56 and the mean severity score is the mean OHIP-14 score. Based on the responses, the prevalence of impact (the percentage of participants responding as ‘fairly often’ or ‘very often’ to one or more items) was also determined. Given the significant skewness of the distribution of OHIP-14 scores, the subjects' OHIP-14 scores were dichotomized as less than or greater than the median value for the studied population, with OHIP-14 scores greater than the median score representing a poor OHRQoL^30^.

Chi-square tests and Fisher's exact tests were used where appropriate to determine associations. Variables that were statistically significant in the Chi-square and spearman correlation tests were entered into a logistic regression model to determine the factors that were significantly independently associated with a poor OHRQoL. Results were considered statistically significant if the P-value was < 0.05.

## Results

A total of three hundred and fifty-two (352) subjects from the two study centres (LUTH and UPTH) participated in the study.

### Socio-demographic data

The mean age was 42.5 years ± 9.4(SD), with most subjects (60.8%) being at least 40 years old. About twothirds, 226(64.2%) of the subjects were females. One hundred and sixty-two (46%) subjects were from the Igbo ethnic group in the South east zone. Significant associations were found in the occupation (P<0.001), socioeconomic status (P=0.002), and geopolitical zone (P<0.001) of the two study centres. The socio-demographic characteristics are shown in Table 1.

**Table 1:** Socio-demographic characteristics of PLWHA by the HIV clinics attended

Characteristic	LUTH	UPTH	Total	P-value
	N (%)	N (%)	N (%)	
**Age group**				
≤19	1 (0.4)	2 (2.0)	3 (0.9)	<0.001*
20–39	80 (31.5)	55 (56.1)	135 (38.4)	
40–59	160 (63.0)	40 (40.8)	200 (56.8)	
≥60	13 (5.1)	1 (1.0)	14 (4.0)	
**Gender**				
Female	169(66.5)	57(58.2)	226(64.2)	0.179
Male	85(33.5)	41(41.8)	126(35.8)	
**Educational level**				
≤ Primary	48 (18.9)	18 (18.4)	66 (18.8)	0.378
Secondary/Diploma	139 (54.7)	47 (47.9)	186 (52.8)	
≥ University	67 (26.4)	33 (33.7)	100 (28.4)	
**Occupation (1>3)**				
1	44 (17.3)	46 (46.9)	90 (25.6)	<0.001*
2	186 (73.2)	41 (41.9)	227 (64.5)	
3	24 (9.5)	11 (11.2)	35 (9.9)	
**Socioeconomic** **Status**				
Upper	63 (24.8)	43 (43.8)	106 (30.1)	0.002*
Middle	179 (70.5)	52 (53.1)	231 (65.6)	
Lower	12 (4.7)	3 (3.1)	15 (4.3)	
Geopolitical Zone				
South West	84 (33.1)	5 (5.1)	89 (25.3)	<0.001*
South South	50 (19.7)	34 (34.7)	84 (23.9)	
South East	104 (40.9)	59 (59.2)	162 (46.0)	
North (NW, NC, NE)	16 (6.3)	1 (1.0)	17 (4.8)	

* Statistically significant

### Clinical findings among the PLWHA

Oral lesions associated with HIV/AIDS were observed in 21(5.9%) subjects with linear gingival erythema being the most common oral lesion (1.99%), followed by candidiasis (1.70%). See Figure 1. The mean CD4+ T lymphocyte count was 552.4 ±237.8 cells/mm^3^ (n=140). The DMFT grade was mild in 72(20.5%) and very mild in 52(14.8%). The mean DMFT was 1.8±2.8, while the mean OHI-S was 1.9±1.0.

### Oral health related quality of life (OHRQoL)

The prevalence of impact caused by oral health problems measured by the OHIP-14 in the preceding 12 months was 8.5% among the PLWHA.

The mean(±SD) and median(range) OHIP-14 scores were 8.05±9.54 and 5.0(0–56), respectively.

Table 2 shows the prevalence of impact and the mean severity score for each of the OHIP-14 item. The prevalence of impact was highest for the “painful aching” item where 67(19.1%) of the subjects reported that they had an impact within the last one year. The other common oral health impact was caused by uncomfortable chewing (17.1%) and avoidance of certain foods (10.8%). With regard to mean domain scores, the highest mean impact was obtained in the domain of physical pain with an overall mean impact of 2.32.

**Table 2:** Prevalence of oral health impact and mean severity score for each item

OHIP domain and items	Frequency (%)	Mean severity score (SD)
**Functional limitation**		
1.Trouble pronouncing words	23(6.6)	0.41(0.97)
2.Worsened taste	25(7.1)	0.51(1.03)
**Physical pain**		
3. Painful aching	67(19.1)	1.28(1.37)
4. Uncomfortable chewing	60(17.1)	1.04(1.36)
**Physical disability**		
5. Diet unsatisfactory	28(8.0)	0.53(1.04)
6. Avoidance of certain foods	38(10.8)	0.60(1.18)
**Psychological discomfort**		
7. Self-conscious	37(10.5)	0.67(1.15)
8. Uncomfortable about	28(8.0)	0.62(1.11)
appearance		
**Psychological disability**		
9. Difficult to relax	32(9.1)	0.65(1.14)
10. Been embarrassed	19(5.4)	0.40(0.93)
**Social disability**		
11. Irritable with others	15(4.3)	0.32(0.84)
12. Difficulty with daily	16(4.5)	0.30(0.81)
activities		
**Handicap**		
13. Unable to function	16(4.6)	0.37(0.87)
14. Life less satisfying	14(4.0)	0.28(0.80)

About a quarter of the subjects 87(24.7%) had a self-reported medical condition with their HIV status, although there was no significant association with the OHRQoL (P=0.733). Subjects with less/good impact of the OHRQoL were 183(52%) while those with poor OHRQoL were 169(48%). Poor OHRQoL was associated with socioeconomic status (P=0.028) and the use of HAART (P=0.020). (Table 3).

**Table 3:** Association of socio-demographic characteristics with poor OHRQoL

	Good OHRQoL	Poor OHRQoL	Total	
			
	N (%)	N (%)	N (%)	P-value
**Age group**				
≤19	1(33.3)	2(66.7)	3(0.9)	0.804
20–39	73(54.1)	62(45.9)	135(38.4)	
40–59	101(50.5)	99(49.5)	200(56.8)	
≤60	8(57.1)	6(42.9)	14(4.0)	
**Gender**				
Male	68(54.0)	58(46.0)	126(35.8)	0.656
Female	115(50.9)	111(49.1)	226(64.2)	
**Socio-economic status**				
Upper	45(42.5)	61(57.5)	106(30.1)	0.028*
Middle	132(57.1)	99(42.9)	231(65.6)	
Lower	6(40.0)	9(60.0)	15(4.3)	
**Period of living with HIV**				
> 10 Years	52(50.0)	52(50.0)	104(29.5)	0.714
≤10 Years	131(52.8)	117(47.2)	248(70.5)	
**Patients on HAART**				
No	28(70.0)	12(30.0)	40(11.4)	0.020*
Yes	155(49.7)	157(50.3)	312(88.6)	
**Self-reported medical conditions**				
No	139(52.5)	110(48.5)	265(75.3)	0.733
Yes	44(50.6)	43(49.4)	87(24.7)	

* Statistically significant

### Self-rated oral health, Self-reported oral problems, medical conditions and HAART use in PLWHA in Nigeria

The subjects' self-rated oral health was excellent in 18.5%, good in 52.8%, fair (26.1%), and poor (2.6%). A significant proportion (68.7%) reported an oral problem in the past one year. Only 88(25%) had utilized the dental clinic in the preceding year, although more than half, 217(61.6%) expressed a self-perceived need for dental treatment. Overall, only about a third 130(30.6%) of the subjects had visited the dentist before while 222(63.1%) had never visited. Majority, 312(88.6%) were on Highly active anti-retroviral therapy (HAART). Self-reported oral problems were reported in 222(71.2%) patients on HAART and 20(50%) of those who were not on HAART. [P=0.029]. The OHRQoL was significantly poor among those with self-reported oral problems (P<0.001), perceived need for dental treatment (P<0.001), dental visit in the past year (P<0.001), presence of oral lesions (P=0.021) and DMFT (P<0.005)

Eighty-six respondents (25.4%) gave a history of an underlying medical conditions. (Table 4).

**Table 4:** Association of poor OHRQoL with oral hygiene practices and clinical findings

	Good OHRQoL	Poor OHRQoL	Total N (%)	P-value
			
	N (%)	N (%)		
**Self-reported oral problems in the last one year**				
Toothache	15(28.8)	37(71.2)	52(14.8)	<0.001*
Swelling of the mouth/face	3(42.9)	4(57.1)	7(2.0)	
Sensitivity to hot/cold drinks	23(60.5)	15(39.5)	38(10.8)	
Mouth Ulcer	4(66.7)	2(33.3)	6(1.7)	
Mouth Odour	10(47.6)	11(52.4)	21(6.0)	
Mobile Teeth	1(16.7)	5(83.3)	6(1.7)	
Hole in Tooth	22(28.2)	56(71.8)	78(22.2)	
Bleeding Gums	18(52.9)	16(47.1)	34(9.7	
Total Oral problems	96(39.7)	146(60.3)	242(68.7)	
None	87(79.1)	23(20.9)	110(31.3)	
**Perceived need of dental treatment**				
No	105(77.8)	30(22.2)	135(38.4)	<0.001*
Yes	78(35.9)	139(64.1)	217(61.6)	
**Dental visit in the past year**				
No	153(58.0)	111(42.0)	264(75.0)	<0.001*
Yes	30(34.1)	58(65.9)	88(25.0)	
**Presence of Oral lesions**				
Hyperpigmentation	1(25.0)	3(75.0)	4(1.1)	0.021*
NUG	0(0.0)	1(100.0)	1(0.3)	
Apthous Ulcer	0(0.0)	3(100.0)	3(0.9)	
LGE	6(85.7)	1(14.3)	7(2.0)	
Candidiasis	5(83.3)	1(16.7)	6(1.7)	
None	171(51.7)	160(48.3)	331(94.0)	
**OHIS Grade**				
Good	43(45.7)	51(54.3)	94(26.7)	0.268
Fair	107(53.0)	95(47.0)	202(57.4)	
Poor	33(58.9)	23(41.1)	56(15.9)	
**DMFT Grade**				
No Caries	103(61.3)	65(38.7)	168(47.7)	0.005*
Very Mild	23(44.2)	29(55.8)	52(14.8)	
Mild	36(50.0)	36(50.0)	72(20.5)	
Moderate	12(40.0)	18(60.0)	30(8.5)	
Severe	9(30.0)	21(70.0)	30(8.5)	

* Statistically significant

In the logistic regression model, all the variables that were statistically significant in the Chi-square and spearman correlation tests were entered into a regression model to determine the factors that were independently associated with poor OHRQoL after controlling for potential confounders were. The independent factors were the perceived need for dental treatment (p<0.001; OR:95%C.I:4.774), use of HAART (p=0.037; OR:95%C.I:2.392), and higher DMFT (p=0.038; OR: 95%C.I: 1.226). Subjects with a perceived need of dental treatment had a higher Odds 4.77(2.79 – 8.16) of reporting a poor OHRQoL. (Table 5)

**Table 5:** Logistic regression model of factors associated with a poor OHRQoL

Independent variables	Adjusted OR	P-value	95% C.I for OR	

			Lower	Upper
SES	0.804	0.094	0.623	1.038
Perceived need for dental treatment	4.716	0.000*	2.783	7.989
Dental visit in the past year	1.325	0.355	0.729	2.408
Presence of oral problems in the last one year	0.888	0.005*	0.817	0.965
Presence of oral lesions associated with HIV/AIDS	0.773	0.600	0.295	2.025
Patients on HAART	2.392	0.037*	1.053	5.432
DMFT Grade	1.226	0.038*	1.012	1.486
Constant	0.465	0.204		

* Statistically significant

SES= Socio-economic status, OR = Odds ratio, at 95% C.I= Confidence Interval

## Discussion

The human immunodeficiency virus infection has remained a devastating disease of public health importance with an impact on the oral health of people living with HIV/AIDS (PLWHA). The present study determined the association between oral health and the quality of life and the factors affecting OHRQoL among HIV positive patients in Nigeria. This was in order to facilitate improved provision of dental care for such individuals.

The study had some limitations such as the convenience sample that was used which might have encouraged the participation of individuals that were more concerned about their oral health, hence may not be fully representative of all PLWHA in Nigeria. The cross-sectional design of the study also makes it difficult to establish a cause-effect relationship between oral health and quality of life. Also, the self-reporting of some data in the study may have recall bias and affected the accuracy of the responses. Nevertheless, the study had many strengths. To the best of our knowledge, it is the first published study on the oral health related quality of life in PLWHA in Nigeria. Furthermore, this study has an ethnic diversity, a relatively large sample from two HIV treatment centres in Nigeria, which gives a broad understanding of the important associations between oral health and the health related quality of life among PLWHA in Nigeria.

The low prevalence of oral health impact on the quality of life of PLWHA in the present study contrasts when compared with other studies^6^,^31^. A higher prevalence of 62.6% was reported among low-income HIV positive adults in the US^6^ and a prevalence of 33.9% was reported among HIV patients in Malaysia^31^. The differences observed between our study and theirs may stem from the difference in the socioeconomic status (SES), as most of the subjects in the two study centres in the present study belonged to the upper and middle socioeconomic class and this may account for the low prevalence of oral impact in the present study. The negative impact of SES on the quality of life of HIV/AIDS patients has been documented previously^32^. The observation of physical pain (“painful aching” and” uncomfortable chewing”) as the most prevalent oral health impact in the present study, may be due to the high proportion of self-reported oral problems by the patients namely toothache, swelling from the mouth/face, mobile teeth and hole in the teeth which are often symptomatic oral conditions. This is comparable to the finding in the Portuguese study by Santo et al,^33^ in which majority of the respondents also reported an impact in the domain of physical pain and this was attributed to their discomfort while eating food. Our finding is however in contrast to the Malaysian study^31^ in which the most affected oral health domain was psychological discomfort. In that study, the most common impact reported was the feeling of discomfort due to food getting stuck in between teeth or dentures.

The use of HAART has been reported to significantly reduce the prevalence of oral lesions and opportunistic illnesses associated with HIV and mortality^34^. This is consistent with the low prevalence of oral lesions (5.9%) in the present study which however contrasts with the South African study in which more than 50% of the PLWHA had two or more oral lesions^22^. However, none of the participants in the South African study was on antiretroviral treatment unlike the present study where majority were on HAART. It has been reported that PLWHA with oral lesions have significantly poor OHRQoL than PLWHA without oral lesions^35^ and may negatively impact the OHRQoL^30^. Oral lesions can alter facial appearance, speech, causing chewing, swallowing difficulty and pain^36^. Thus, poor oral function will exacerbate nutritional problems that may further affect their quality of life^35^,^36^.

Despite the low prevalence of oral lesions in the present study, HAART use by patients was independently associated with poor OHRQoL which was baffling to us. This raises some valid concerns about some potential side effects of HAART on oral health in the course of managing HIV/AIDS infection. Whilst, HAART is beneficial in reducing the prevalence of oral lesions, the type of HAART administered to patients could also, influence the OHRQoL. This may be influenced by the reduced immunologic response and decreased salivary function with the continuous use of the medications. Again, the reason for the finding may not be too farfetched considering the significantly higher prevalence of self-reported oral problems in patients that were on HAART (71.2%) compared to those not on HAART (50%). The presence of painful oral lesions and conditions can contribute to ineffective or infrequent tooth brushing leading to discomfort and disability, masticatory inefficiency and consequently the OHRQoL.

The prevalence of dental caries in the present study was relatively low (mean DMFT of 1.8) when compared to other studies in HIV positive populations where the mean DMFT varied from 8.7 in Australia^7^ to 16.9 in Portugal^33^ and 18.8 in Brazil^37^. This may be reflective of the low prevalence of dental caries in the general Nigerian population^38^. The low DMFT in this study could also be attributed to the relatively good oral hygiene habits by the subjects as more than half of them had a fair oral hygiene status. Nevertheless, the unmet need from the untreated carious lesions is still somewhat a course for concern because higher DMFT scores may be reflective of higher morbidity in terms of pain and/or swelling.

The significantly poorer OHRQoL among those with self-perceived need of dental treatment is consistent with previous studies^22^,^31^. The awareness of oral health problems and the need to access treatment may be seen by the patients to have an impact on their overall health. Although, about two thirds (61.4%) of the patients reported at least one dental problem that needed professional attention, only about half had actually visited the dentist. This reflects an unmet need among the PLWHA and their inadequate utilization of dental services which buttresses the fact that having dental issues alone is not sufficient reason for an individual to visit the dental clinic. There are other factors that could serve as potential barriers to accessing care such as finances and unavailability of clinics which were among the factors highlighted in our previous study^39^.

In agreement with previous studies^22^,^40^, this study identified more females compared to males. Women have been reported to be more expressive and concerned about their oral health than men as it causes them pain and embarrassment^40^. In addition, females are more likely to relate their oral health to their appearance and quality of life.

## Conclusion

Within the limitations earlier highlighted in this study, the domain of physical pain had the highest oral health impact, while the perceived need for dental treatment, HAART use and higher caries index were contributory to poor OHRQoL. Our findings highlight the importance of routine dental check-up for PLWHA particularly among those on HAART and the role of dentists in their interdisciplinary management.
